# Comparison of basophil activation testing and component-resolved diagnosis in patients with cross-reactive intradermal results after anaphylactic reactions to hymenoptera venom

**DOI:** 10.1038/s41598-025-18601-x

**Published:** 2025-11-13

**Authors:** Mohammad Hasan Bemanian, Negin Jafariaghdam, Faezeh Shahba, Mohammad Nabavi, Morteza Fallahpour, Sima Shokri, Fatemeh Faraji, Mohammad-Ali Assarehzadegan, Mehrnaz Mesdaghi, Hamideh Nodehi, Majid Khoshmirsafa

**Affiliations:** 1 Department of Allergy and Clinical Immunology, Rasool-e-Akram Hospital, Iran University of Medical Sciences, Tehran, 49179 37120 Iran; 2 Immunology Research Center, Institute of Immunology and Infectious Diseases, Iran University of Medical Sciences, Tehran, 14496 14535 Iran; 3 Department of Immunology, School of Public Health, Tehran University of Medical Sciences, Tehran, Iran; 4 Department of Immunology, School of Medicine, Iran University of Medical Sciences, Tehran, Iran; 5 Antimicrobial Resistance Research Center, Institute of Immunology and Infectious Diseases, Iran University of Medical Sciences, Tehran, Iran; 6 Department of Allergy and Clinical Immunology, Mofid Children’s Hospital, Shahid Beheshti University of Medical Sciences, Tehran, Iran

**Keywords:** Basophil activation test, Hymenoptera venom allergy, Venom immunotherapy, Component-resolved diagnosis, Cross reactivity, Immunology, Immunotherapy

## Abstract

Hymenoptera venom allergy (HVA) is a significant cause of anaphylaxis, with a broad spectrum of symptoms. Accurate diagnosis is critical for selecting appropriate venom immunotherapy (VIT), yet multiple sensitizations complicate the identification of the culprit Hymenoptera. Component-resolved diagnostics (CRD) and the basophil activation test (BAT) are emerging approaches that may enhance the precision of HVA diagnosis. This study aimed to compare the outcomes of the BAT and CRD tests in patients with reaction to more than one type of Hymenoptera in intradermal test. Following 12 patient’s report-based determination of the Hymenoptera types, Intradermal testing with venom extracts from honey bees, paper wasps, and yellow jackets was performed. CRD was conducted using recombinant allergens for each venom, and BAT was performed to assess basophil activation using venom extracts. The intradermal test indicated 10 triple-positive reactions (83.3%) and 2 double-positive reactions (16.7%). In addition, 7 (58.4%) individuals were triple-positive and 5 (41.6%) were double-positive in the CRD test. The BAT had 6 (50.0%) double-positive individuals and 3 (25.0%) single-positive patients. The diagnostic complexity in patients with multi-sensitivity to Hymenoptera venoms is highlighted by the results. While CRD provides a more specific identification of allergenic components, valuable information is offered by the BAT, especially when the clinical diagnosis is uncertain. As a result, relying just on one test seems to have limited diagnostic capabilities and it is more efficient to use many diagnostic tests in order to get a thorough evaluation.

**Supplementary Information:**

The online version contains supplementary material available at 10.1038/s41598-025-18601-x.

## Introduction

Hymenoptera venom allergy (HVA) is well recognized as a significant etiological factor for anaphylaxis in humans. The immune response following a sting may range from a localized reaction to a broad spectrum of systemic symptoms, affecting the integumentary, respiratory, cardiovascular, gastrointestinal, and even neurological systems^1^. HVA is thereby a potentially life-threatening allergy, requiring a comprehensive diagnosis for patients with systemic symptoms^2,3^. The optimal therapeutic approach for managing venom allergies is presently considered to be venom immunotherapy (VIT), which is effectively administered and executed through precise diagnostic procedures. In addition to enhancing the overall quality of life, VIT can decrease the risk of severe reactions^4^.

The main disadvantage of skin tests for HVA, as conventional tests for allergies, is that they are performed using venom extracts^5^. According to the studies, among the most common allergenic species of the Vespidae family, yellow jackets (Common wasp, Vespula spp.) and paper wasps (Polistes spp.) exhibit significant cross-reactivity, attributed to the presence of 48 shared venom proteins^6^. Also, double positivity for the venoms of the wasps from the Vespidae family and honey bees from the Apidae family has been indicated in patients with HVA^7^. Despite the significant structural differences between the venoms of honey bees, yellow jackets, and paper wasps, they share common proteins such as phospholipases (Api m 1, Ves v 1, and Pol d 1 for honey bee, yellow jacket, and paper wasp, respectively), hyaluronidases (Api m 2, Ves v 2, and Pol d 2 for the same species, respectively), and cross-reactive carbohydrate determinants (CCDs). These shared components contribute to cross-reactivity in individuals allergic to the venom of any of these Hymenoptera^8^. Consequently, multiple sensitizations often complicate the identification of the specific culprit Hymenoptera species, hindering the selection of the most appropriate VIT^9^.

Component-resolved diagnostics (CRD) is an immunoassay that measures specific IgE antibodies directed against individual allergenic molecules using a microarray platform containing recombinant venom allergens; by utilizing recombinant allergens and minimizing the influence of cross-reactive carbohydrate determinants (CCDs), CRD offers a more precise diagnostic approach for distinguishing between venom sensitivities^10^. Another promising diagnostic approach is the Basophil activation test (BAT), an in vitro assay that measures basophil activation via flow cytometry, and offers high specificity as it directly reflects histamine release and IgE-mediated reactions^11^. It has been highlighted recently that the BAT’s value increases when combined with other diagnostic tests^12^.

The objective of the present study was to compare the diagnostic performance of BAT and CRD in patients with HVA who demonstrated at least two positive reactions to honey bee, yellow jacket, and paper wasp venom extracts during intradermal test.

## Materials and methods

### Patient selection

Patients with a documented history of anaphylaxis to Hymenoptera stings who were referred to the Allergy Clinic of Rasoul Akram Hospital in Tehran, Iran, between March and September 2023, with informed consent to participate, were enrolled in this cross-sectional study. All patients with non-systemic reactions to Hymenoptera stings, anaphylaxis to fire ant venom, and those who had monosensitization in intradermal test or CRD with one of the allergen components were excluded from the study. Every patient included in the study provided a comprehensive medical history, such as associated comorbidities, medication list, reaction severity, family history of allergies and anaphylaxis, and the date of the last anaphylactic reaction. All patients had a refractory period of at least 4 weeks from their last anaphylactic attack and discontinued antihistamines and drugs that could interfere with test results at least 1 week before testing.

### Intradermal test

Following the patient’s self-report-based identification of the culprit Hymenoptera, the intradermal test using commercial venom extracts for the honey bee, paper wasp, and yellow jacket (Jubilant Hollister Stier LLC, WA, and USA) was performed during the initial evaluation. The intradermal test was performed in the forearm area through 0.02 ml of a 1 µg/ml dilution of venom extracts^13^. Histamine and saline were used as positive and negative controls, respectively. The reactions were evaluated 15 min after the test, and a wheal more than 3 mm was considered positive.

### Component-resolved diagnosis

In cases in which the intradermal test yielded positive for more than one type of Hymenoptera, the corresponding CRD test was performed using the Macroarray Alex, Austria kit, and Api m allergens (Api m 1, Api m 2, and Api m 10) for honey bees, Pol d allergens (Pol d 5) for paper wasps, and Ves V allergens (Ves v 1 and Ves v 5) for yellow jacket. According to the kit’s instructions, after removing the required number of cartridges, they were placed in the holder. Following the addition of 100 µL of the patient samples and 400 µL of sample diluent, the cartridges were incubated at 8 rpm for 2 h. The washing procedure commenced with adding 500 µl of washing solution to each cartridge and placing them on a rocker (at a speed of 8 rpm) for 5 min. Then the washing solution was drained and dried by tapping the cartridges vigorously on paper towels. Right after, 500 µL of detection antibody was added to each cartridge. The washing was repeated as in the previous washing step. In the next step, by adding 500 µL of substrate and washing as before, but this time for 30 s, the assay procedure was completed and scanned with ImageXplorer. Quantitative data acquisition and signal analysis were performed using the RAPTOR SERVER Analysis software (version 1.3; Macro Array Diagnostics GmbH, Vienna, Austria; https://www.macroarraydx.com).

### Basophil activation test

For cases that were positive for more than one type of Hymenoptera in the intradermal test, in addition to CRD, the BAT was performed using the BasoFlowEx kit (EXBIO, Vestec, Czech Republic) to identify the culprit Hymenoptera.

Following the instructions of the kit, 100 µL of heparinized whole blood was incubated with 100 µL of stimulation buffer and 15 µL of allergen, which were 1 µg/ml venom extracts of honey bee, paper wasp, and yellow jacket, for 20 min at 37 °C. Each determination was evaluated in parallel with a positive and negative control. The positive control was incubated with 100 µL of stimulation buffer and 15 µL of the stimulation control, which contained the chemotactic peptide N-formyl-Met-Leu-Phe (fMLP). The negative control contained only the stimulation buffer.

After incubation, 20 µL of staining reagent containing anti-CD203c-PE (clone NP4D6) for basophil population identification and anti-CD63-FITC (clone MEM-259) for measuring basophil activation was added to each sample, followed by a 20-minute incubation at 4 °C.

Subsequently, the samples were incubated with 300 µl of lysing solution for 5 min at room temperature and 2.5 ml of demineralized water for 10–15 min at room temperature. Finally, flow cytometry analysis was performed using Sysmex (CyFlow^®^ Space, Germany) within 2 h after staining.

### Flow cytometry gating strategy

**Fig. 1:**
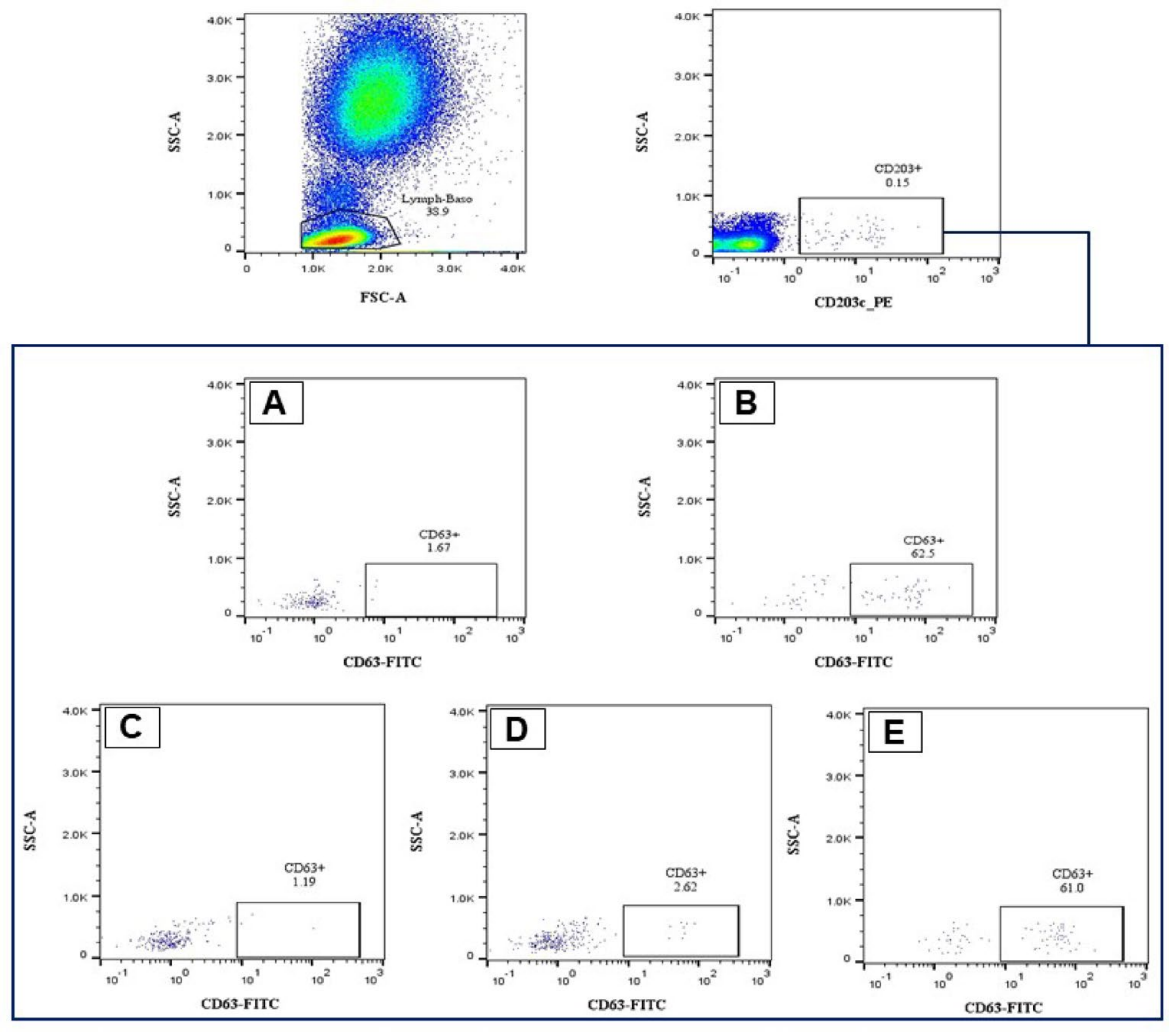
Gating strategy of flow cytometry. By comparing forward scatter to side scatter, the lymphocyte/basophil fraction was initially chosen. As basophils, side scatter-low and CD203c-high cell populations were gated. Activated and degranulated basophils were ultimately selected by separating the populations of cells with low side scatter and high CD63. The activation of more than 10% of basophils was regarded as an affirmative outcome of the BAT (A: Positive control, B: Negative control, C: Honey bee, D: Paper wasp, E: Yellow jacket).

In order to evaluate a sufficient number of basophils (> 200), 50,000–100,000 events per sample were acquired, and the appropriate gate was selected for the population of basophils, which are located in the lymphocyte population. The lymphocyte/basophil fraction was first selected by gating side scatter vs. forward scatter. Eventually, activated and degranulated basophils were selected by gating the side scatter-low and CD63-high cell populations. A positive result of the BAT was considered when more than 6% of basophils were activated (Fig. 1).

### Statistical analysis

Statistical analyses were performed using FlowJo 10.8, SPSS version 26.0 (SPSS Inc., Chicago, Illinois, USA), and GraphPad Prism version 9.0 (GraphPad, La Jolla, California) for presenting the results. The interpretation of the BAT included setting up the cut-off level, which was determined by the mean of the negative control results and the sensitivity index (SI) by three times the standard deviation. According to this criterion, any results below 6.0% were considered negative, and any results above 6.0 were considered positive. Also gating strategy has been set according to the EAACI protocol^14^.

## Results

### Demographic and clinical characteristics

Among the 12 patients with anaphylaxis to various types of Hymenoptera venom enrolled in this study, 10 (83.3%) were male, and 2 (16.7%) were female. The mean ± SD age of patients participating in the study was 26.5 ± 17.85 years. Table 1 provides a summary of the demographic characteristics, including patients’ histories, the suspected Hymenoptera species responsible for the reactions, the severity of anaphylaxis, history of atopy, and anaphylaxis in first-degree family.

**Table 1:** Demographic and clinical characteristics of enrolled patients.

Gender	Culprit Hymenoptera^*^	Anaphylaxis grade	History of atopy	Family history
Female: 2 (16.7%)	Honey bee: 4 (33.3%)	Grade 3: 4 (33.3%)	3 (25%) with combined history of conjunctivitis and seasonal allergic rhinitis	1 (8.4%)
	Yellow jacket: 3 (25.0%)			
Male: 10 (83.3%)	Paper wasp: 4 (33.3%)	Grade 4: 8 (66.7%)		
	Unknown: 1 (8.4%)			

The patients had underlying medical conditions such as hypertension, hyperlipidemia, seizures, and anxiety disorders. Their medications included aspirin, Depakine, topiramate, citalopram, and atorvastatin.

### Intradermal test

**Fig. 2:**
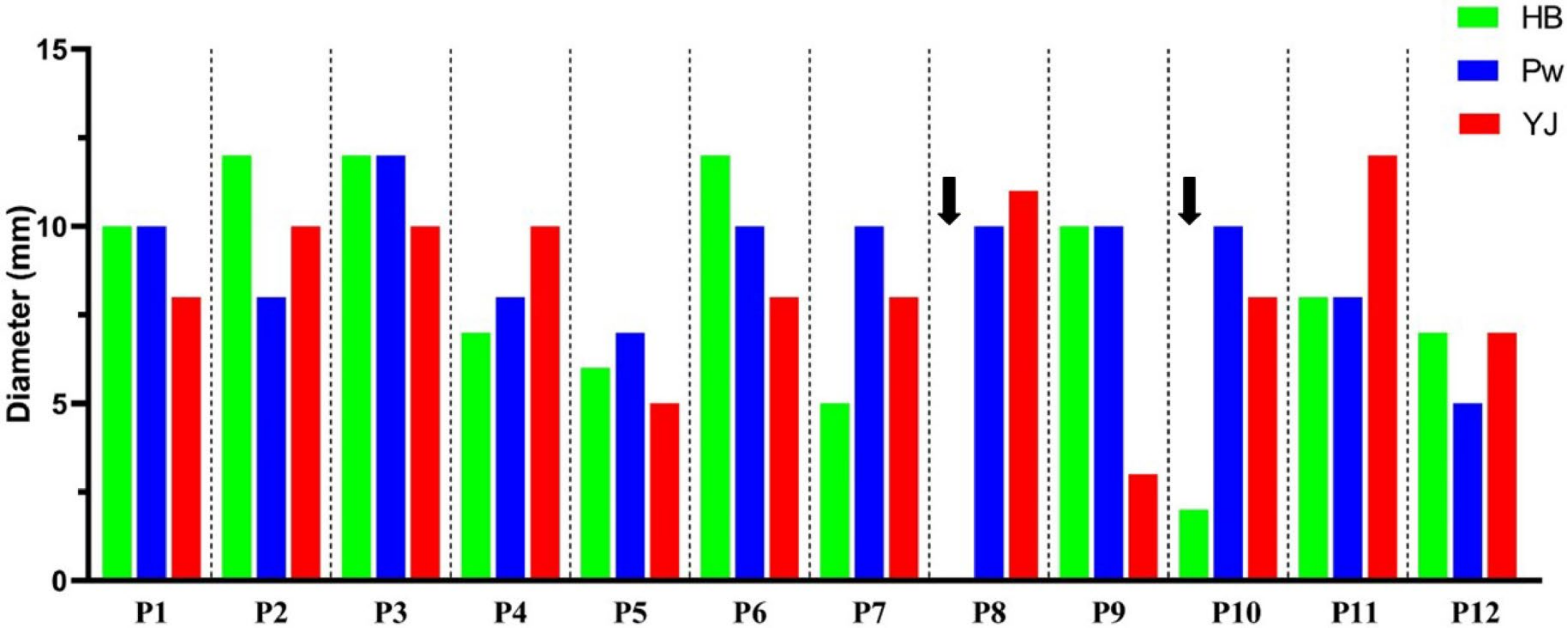
Results of intradermal test of patients by type of hymenoptera (HB: Honey bee, YJ: Yellow jacket, PW: Paper wasp).

In intradermal test with the extracts of three Hymenoptera, 10 of 12 patients (83.3%) with a 7.58 ± 3.91 mm and a range of 2–12 mm wheal were positive for honey bee. All patients had a positive result for paper wasp. The mean ± SD wheal created was 9 ± 1.85 mm and its range was between 5 and 12 mm. Also, the result of intradermal test for yellow jacket was positive for all patients. The mean ± SD wheal created was 8.33 ± 2.53 mm, and its range was measured between 3 and 12 mm (Fig. 2). The outcomes of the intradermal test for each patient are presented in Supplementary Fig. 1.

### Component-resolved diagnosis

In the CRD test, 9 patients (75.0%) had positive results for at least one component of the honey bee, 11 patients (91.7%) had positive results for at least one component of paper wasp, and 11 (91.7%) had positive results for at least one component of yellow jacket. The mean sIgE obtained to Api m was 8.42, and its range was between 0.36 and 30.23. The mean sIgE obtained to Api m 1 was 5.7, and its range was between 0.35 and 28.49. Also, the mean sIgE obtained to Api m 10 was 15.29, and its range was 2.36–32.30. For Pol d, the range of sIgE values was 0.31–4.67, with a mean of 1.77. Pol d 5 received a mean sIgE of 3.99 with a range of 0.59–10.91. Also, the mean sIgE obtained in Ves v was 1.01, and its range was 0.24–3.34. sIgE ranged from 0.84 to 3.45, with a mean of 2.06 obtained for Ves v 1. The mean number of sIgE obtained to Ves v 5 was 2.98, and its range was between 0.87 and 7.45 (Supplementary Table 1). Except for five cases, all patients had positive CRD results for all three types of Hymenoptera venom; among these 5, 3 had positive results for yellow jacket and paper wasp, 1 had positive results for honey bee and yellow jacket, and 1 had positive results for honey bee and paper wasp (Table 2).

**Table 2:**
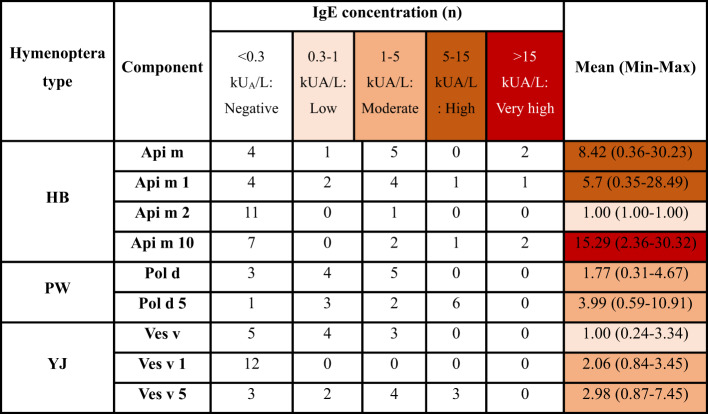
Results of CRD tests for patients, classified by hymenoptera species and component (HB: Honey bee, PW: Paper wasp, YJ: Yellow jacket, BAT: Basophil activation test, CRD: Component resolved diagnosis, <0.3: Negative (0), 0.3–1: Low IgE Level (1), 1–5: Moderate (2), 5–15: High IgE Level (3), >15: Very high IgE Level (4)).

### Basophil activation test

According to the results of BAT, two patients (16.7%) were positive for paper wasp, and a patient (8.4%) was positive for yellow jacket. Moreover, a patient (8.4%) was double-positive for honey bee and yellow jacket, and five patients (41.7%) were double-positive for paper wasp and yellow jacket. Considering that patient No. 6 did not respond to the positive control, it was excluded from the results for analysis. The outcomes of the other two patients with negative results for all three types of Hymenoptera were valid because they responded to the positive control.

## Discussion

The present study included 12 individuals with a history of anaphylactic reactions to at least two Hymenoptera venoms, including honey bee, yellow jacket and/or paper wasp. Based on the findings, there were 10 patients (83.3%) with a triple-positive and 2 patients (16.7%) with a double-positive reaction for intradermal test, 7 patients (58.4%) with a triple-positive and 5 patients (41.7%) with a double-positive reaction in CRD test, and 6 patients (50.0%) with double-positive and 3 patients (25.0%) with single-positive reaction in the BAT.

**Fig. 3:**
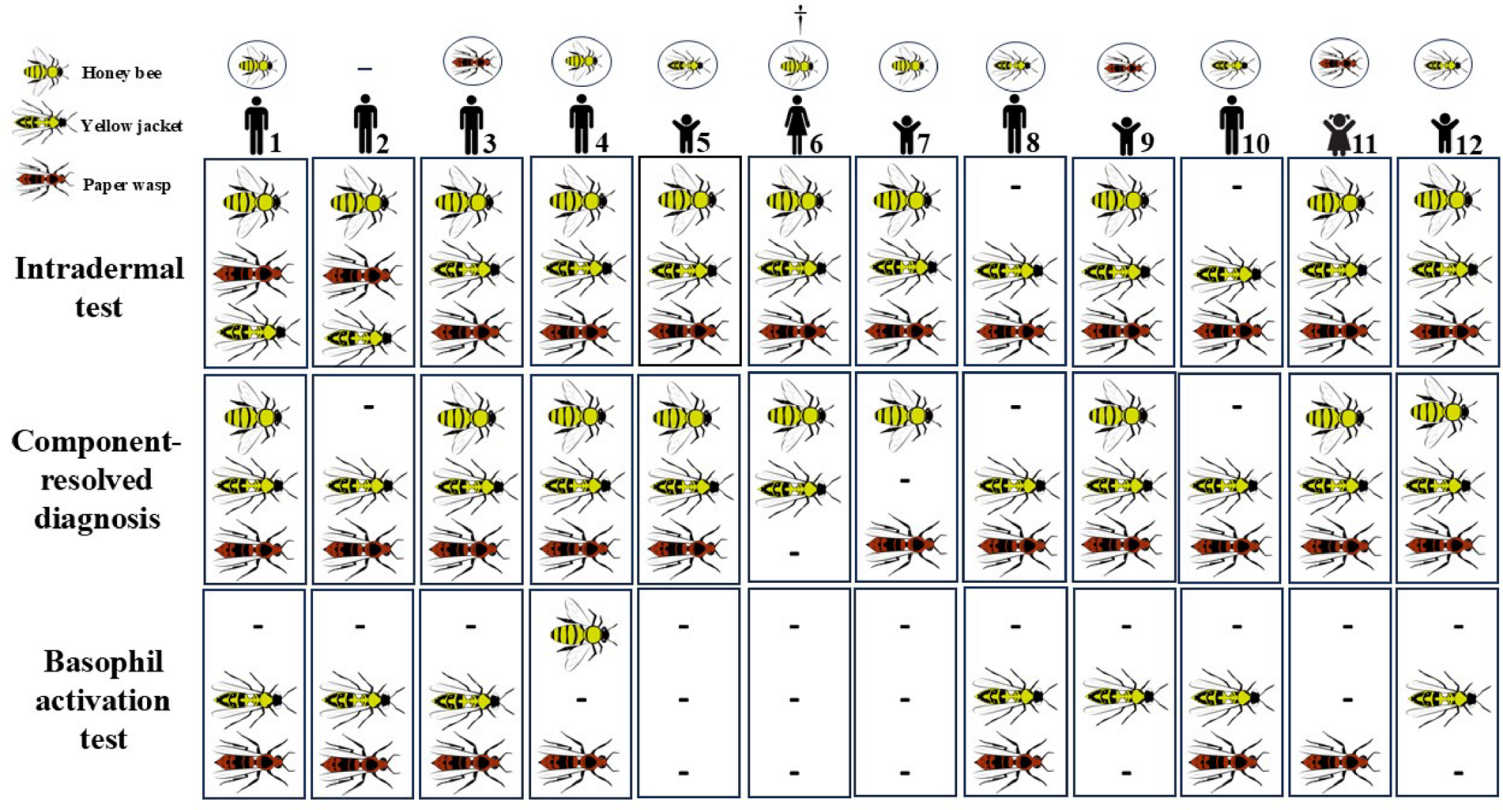
Comparison of the outcomes of intradermal test, CRD test, and BAT in all patients (†: Patient No.6 did not respond to the positive control, so it was excluded from the results of the BAT for analysis).

Comparing these three tests, it was found that the outcomes of six patients (50.0%) who were triple-positive in the intradermal test remained unchanged in the CRD test, whereas there was no triple-positive reaction in BAT. Also, two patients were double-positive with reactions to paper wasp and yellow jacket in the intradermal test, which showed the exact same result in both the CRD test and the BAT. So, it can be concluded that only three out of all patients (25.0%) were found to have similar results between three tests performed in the present study. In addition, it can be mentioned that among seven patients who were triple-positive in both intradermal and CRD tests, 3 (42.8%) of them turned to double-positive, 3 (42.8%) of them turned to single-positive, and 1 (14.4%) of them showed a negative result in BAT. Furthermore, among two patients who were triple-positive in the intradermal test and turned double-positive in CRD, a patient remained double-positive, but another patient showed a negative result in BAT (Fig. 3, Table 3).

**Table 3:**
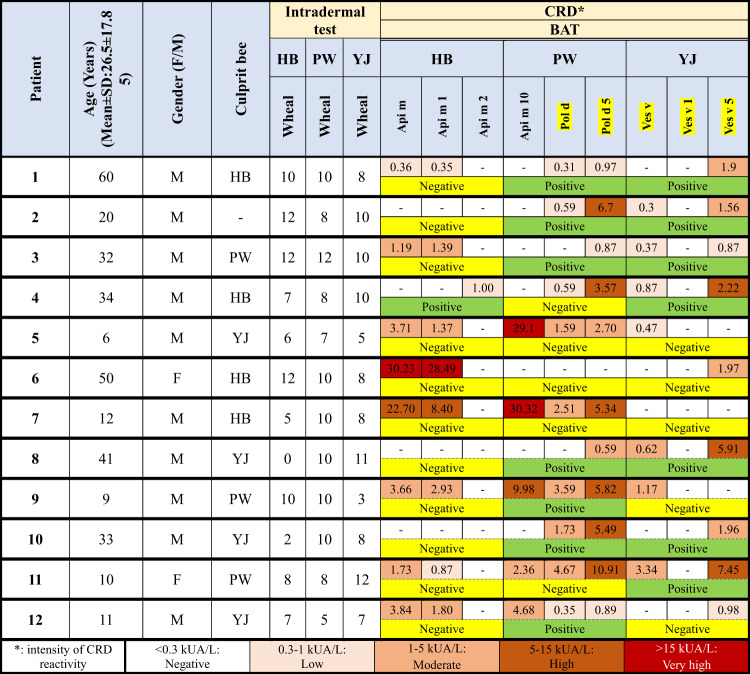
The details on the outcomes of skin tests, CRD test, and BAT in patients (HB: Honey bee, PW: Paper wasp, YJ: Yellow jacket, BAT: Basophil activation test, CRD: Component resolved diagnosis, W/E: Wheal/Erythema).

The Vespidae subfamilies are well-established allergenic wasp groups that, despite being taxonomically distinct from bees of the Apoidea superfamily, exhibit a significant degree of cross-reactivity. This immunological overlap is primarily attributed to structural similarities in venom components, which are recognized by specific IgE antibodies. As a result, distinguishing between true sensitization and cross-reactivity can be diagnostically challenging^15^. Accurate identification of the culprit Hymenoptera species is therefore crucial, as it reduces the risk of misdiagnosis and ensures the selection of the appropriate venom for immunotherapy, the most effective treatment for Hymenoptera sting-induced anaphylaxis^16^. In clinical practice, identification of the culprit Hymenoptera requires an extensive medical record combined with quantification of venom sIgE antibodies and venom skin tests^17–19^.

Recently, the CRD test has shown efficacy in some research. For example, in a study, the combined use of sIgE antibodies targeting rVes v 1 and rVes v 5 from wasps (Vespula vulgaris) allowed for a valid diagnosis of wasp venom allergy in 94% of the 148 patients^20^. In individuals with a honey bee allergy, comparable findings have been reported for a combination of rApi m 1, rApi m 2, rApi m 3, native Api m 4, rApi m 5, and rApi m 10^21^. However, even though CRD test has generally made targeted immunotherapy more specific, currently there isn’t enough strong clinical data to support this conclusion, and there aren’t any clear cut-off values for CRD that are clinically relevant^22,23^. In our study, it was found that in only three patients, the results of the CRD test were different from the intradermal test. Among patients with triple-positive results, some exhibited low specific IgE concentrations (0.3–1 kUA/L) detected by CRD, including three patients for paper wasp, two for honey bee, and three for yellow jacket. Additionally, very high IgE concentrations (above 15 kUA/L) were observed only for Api m allergens (Api m 1 and Api m 10), suggesting that honey bee venom is more likely the cause of allergic reactions in these individuals compared to other Hymenoptera species.

Moreover, some discrepancies between CRD and extract tests were noted, with some patients positive for specific components yet negative for corresponding extracts. This may result from reduced levels of specific allergens in commercial extracts or loss of extract potency during processing, whereas CRD’s purified recombinant components offer greater sensitivity. Technical and biological factors may also influence these differences.

Previous research has suggested that alternative methods, including the use of BAT in clinical settings for routine diagnostics, the identification of new allergens through venomic analysis, and the characterization of the molecular basis of cross-reactivity, could be implemented to address the substantial limitations and unresolved issues in the molecular diagnostics of HVA^24^. The findings of a prospective investigation showed that the sensitivities of BAT, CAP inhibition, and skin tests are similar (87.7%, 91.2%, and 93.0%, respectively). However, the specificity of BAT is higher than that of specific IgE detection (86.7% vs. 66.7%). They also added that BAT combined with skin tests had a sensitivity of 100%^25^. Furthermore, Korosec et al. investigated a group of 47 individuals who had a compelling medical background of HVA but tested negative for specific IgE. Among them, 37 patients tested with BAT and intradermal skin test, and the BAT showed notably greater diagnostic sensitivity compared to the intradermal test (76% vs. 46%)^26^.

In a 2022 study, Cabrera et al. demonstrated that the BAT was more effective than CAP inhibition in reducing cross-reactivity, with a sensitivity of 91.67% compared to 58.3%. The study also reported a positive agreement of 71.43% between the two methods. Notably, BAT successfully identified all indeterminate results from CAP inhibition, achieving a specificity of 83.3%^17^. Also, Santos et al. in 2021 pointed out that among diagnostic tests, BAT has the highest sensitivity (85–100%) and specificity (83–100%) in diagnosing venom allergy. Although the use of sIgE against the major allergens in bees and wasps, Api m 1 and Ves v 5, has reduced the effectiveness of BAT in a large number of suspected cases, BAT still has the greatest success when solving the unique diagnostic problems of venom allergy^27^. Moreover, it turned out that about one-third of the patients’ information about the clinically relevant Hymenoptera can be obtained by BAT through incubation of cells with extracts of bee and wasp venom. Patients with exclusive monosensitization to vespid venom in the BAT and double sensitization (skin test and particular IgE antibodies) might validate the clinical significance of such BAT findings^28^. Also, Ebo et al. found that for about half of the patients, the specific IgE and venom skin test results are good enough and no other tests are needed. But for the other half, the diagnosis is not so clear because the specific IgE or skin test results are inconsistent or negative. In these cases, BAT is a useful additional tool that can help find the venom and start venom immunotherapy^29^.

On the other hand, there are very limited findings indicating the BAT’s ineffectiveness. For instance, in a study involving seven patients with systemic mastocytosis, a convincing history of venom hypersensitivity, and negative venom skin tests, the BAT was only positive to the negative control in one patient and thus did not provide any useful information^30^.

The striking similarities between Pol d 5 and Ves v 5 may explain why five out of six patients who were double-positive in BAT findings had reactions to paper wasp and yellow jacket. As previously stated, these two components of the paper wasp and yellow jacket are nearly identical, making cross-reactivity between these two antigens likely given that the test was performed using Hymenoptera extracts^31^.

It is also common in patients with HVA to show IgE against yellow jacket and honey bee venom, which might be the result of cross-reactivity or co-sensitization to CCDs in both venoms^6^. Based on the research, the frequency of cross-reactivity is around 50–70% between yellow jacket and paper wasp, 10–15% between honey bee and yellow jacket, and less than 10% between honey bee and paper wasp^8^. According to research by Eberlein et al. in 2012, BAT evaluated with horseradish peroxidase as a CCD sensitizer and had a 92% specificity for detecting basophilic responses to CCD, which may be helpful in identifying those patients who are truly sensitive to more than one venom^32^. In 2010, Mertens et al. sought to answer the question of whether IgE antibodies against CCD have biological activity in the BAT. IgE levels in skin tests, venom, specific antibodies against CCD, and BAT with native venom and venom without CCD were measured in 62 patients with HVA, concluding that CCDs do indeed have biological activity but are probably clinically irrelevant, meaning they do not cause clinical symptoms^33^.

In our study, given that CRD was performed before BAT, all patients were tested for anti-CCD antibodies, and all but one patient was reported to be negative for anti-CCD antibodies. This patient was monosensitized in BAT. Therefore, CCD was excluded as one of the reasons for double or triple positivity in the study population.

Similar to the current research, Bokanovic et al. in 2020 reported some negative results in the BAT. They claimed that since 92% of patients with a negative BAT successfully endured the bee sting challenge test without systemic reactions and 7% had minor systemic reactions, unnecessary specific immunotherapy can be avoided^34^.

As the BAT closely resembles the in vivo pathway leading to symptoms, we believe that the technique might further be adopted to differentiate between clinically relevant and irrelevant IgE antibodies, to study cross-reactivity, and quantitative evaluation of (residual) allergenicity^35^. Furthermore, basophil reactivity has the lowest rate of double positivity of diagnostic tests for Hymenoptera allergy^36^.

Our study found concordant results between BAT and CRD in only three cases. This limited agreement, also reported in previous studies^37,38^, likely arises from the distinct immunological mechanisms assessed by each test. While CRD measures serum-specific IgE concentrations, BAT evaluates ex vivo basophil activation, which depends on multiple biological factors, including receptor expression and cellular responsiveness. This divergence may explain why some individuals with elevated sIgE levels demonstrated negative BAT outcomes.

It is noteworthy that enhancing the BAT method through the use of recombinantly produced CCD-free allergens and conducting the test at varying antigenic concentrations could yield more favorable results. This approach would be beneficial in minimizing the frequency of unnecessary immunotherapy in patients.

## Conclusion

Determining the culprit allergen in anaphylaxis following Hymenoptera stings is a challenging issue. The present research intends to evaluate the efficacy of BAT and CRD tests in reducing multiple-positive results by comparing the outcomes of these tests in patients who had reactions to more than one type of Hymenoptera in the intradermal test. In our study, BAT demonstrated a higher frequency of single-positive cases relative to CRD. Nevertheless, further research is required to assess the diagnostic efficacy of this approach in complex conditions.

## Supplementary Information

Below is the link to the electronic supplementary material.

Supplementary Material 1

Supplementary Material 2

Supplementary Material 3

## Data Availability

Data is provided within the manuscript or supplementary information files.
