# Supplementary material for: Comparison of basophil activation testing and component-resolved diagnosis in patients with cross-reactive intradermal results after anaphylactic reactions to hymenoptera venom
Source: Sci Rep. 2025 Nov 13;15:39751. doi: 10.1038/s41598-025-18601-x (PMC12615578; doi:10.1038/s41598-025-18601-x)
Supplement: Supplementary file 1 — Supplementary Material 1 [file 41598_2025_18601_MOESM1_ESM.docx]

**Supplementary Table 1.** CRD Component frequency and percentage by sIgE level grouping.

| **Ves V5**  **N (%)** | **Ves V1**  **N (%)** | **Vesv**  **N (%)** | **Pold 5**  **N (%)** | **Pold**  **N (%)** | **Api m10**  **N (%)** | **Api m2**  **N (%)** | **Api m1**  **N (%)** | **Api m**  **N (%)** | **sIgE**  **Level**  **IU/ml** |
| --- | --- | --- | --- | --- | --- | --- | --- | --- | --- |
| 2 (17%) | 1 (8%) | 4 (33%) | 4 (33%) | 4 (33%) | 0 (0%) | 0 (0%) | 2 (17%) | 1 (8%) | 0.3-1 |
| 5 (42%) | 3 (25%) | 2 (17%) | 2 (17%) | 5 (42%) | 2 (17%) | 1 (8%) | 4 (33%) | 6 (50%) | 1-5 |
| 2 (17%) | 0 (0%) | 0 (0%) | 5 (42%) | 0 (0%) | 1 (8%) | 0 (0%) | 1 (8%) | 0 (0%) | 5-15 |
| 0 (0%) | 0 (0%) | 0 (0%) | 0 (0%) | 0 (0%) | 2 (17%) | 0 (0%) | 1 (8%) | 2 (17%) | >15 |
| **9 (75%)** | **4** (**33%)** | **6 (50%)** | **11 (92%)** | **9 (75%)** | **5 (42%)** | **1 (8%)** | **8 (66%)** | **9 (75%)** | >0.3 |
