# Supplementary material for: Comparison of basophil activation testing and component-resolved diagnosis in patients with cross-reactive intradermal results after anaphylactic reactions to hymenoptera venom
Source: Sci Rep. 2025 Nov 13;15:39751. doi: 10.1038/s41598-025-18601-x (PMC12615578; doi:10.1038/s41598-025-18601-x)
Supplement: Supplementary file 3 — Supplementary Material 3 [file 41598_2025_18601_MOESM3_ESM.docx]

**
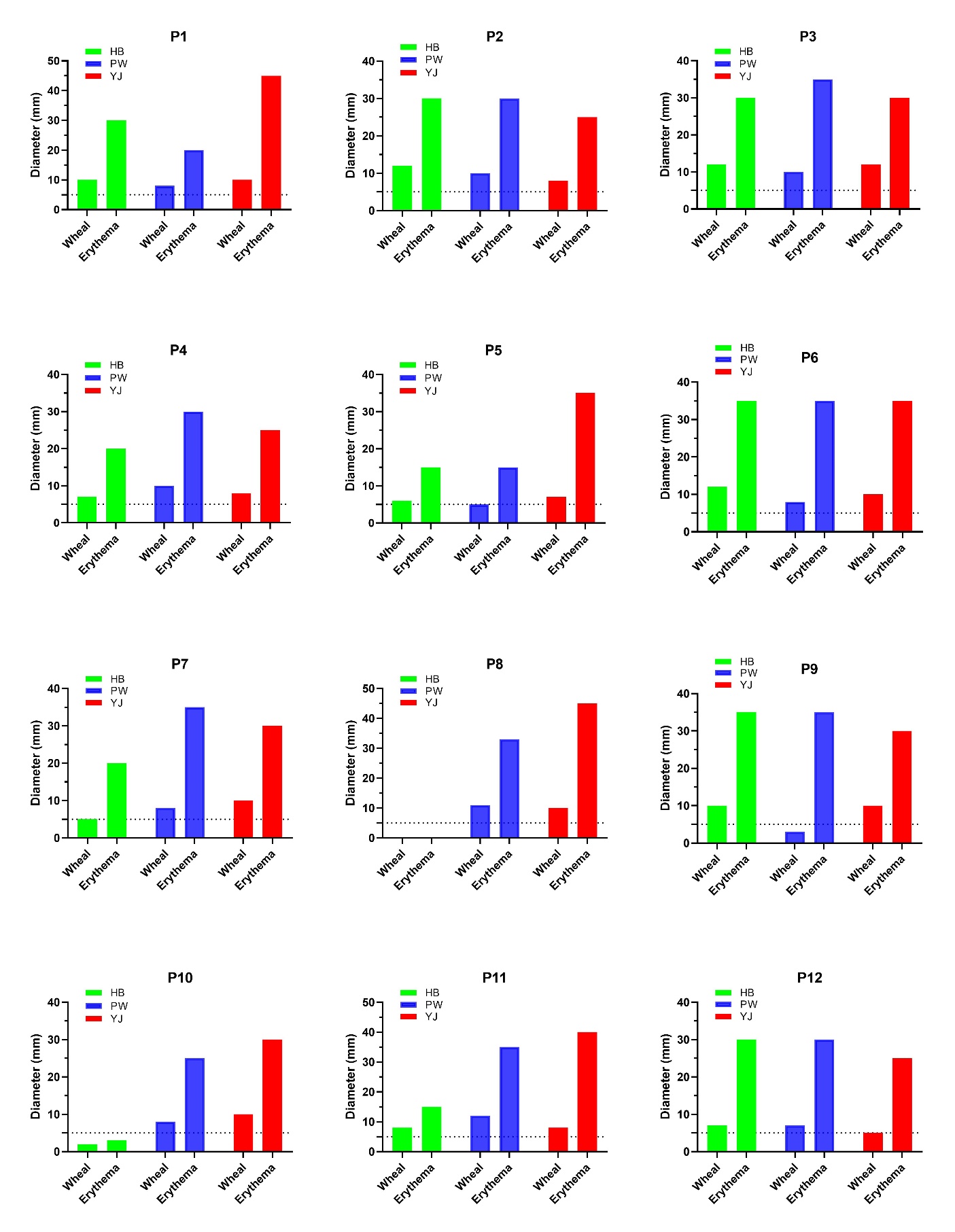
**

**Supplementary Figure 1.** Results of intradermal skin test by type of the hymenoptera for each patient.
